# Enhancing stability of recombinant CHO cells by CRISPR/Cas9-mediated site-specific integration into regions with distinct histone modifications

**DOI:** 10.3389/fbioe.2022.1010719

**Published:** 2022-10-13

**Authors:** Oliver Hertel, Anne Neuss, Tobias Busche, David Brandt, Jörn Kalinowski, Janina Bahnemann, Thomas Noll

**Affiliations:** ^1^ Cell Culture Technology, Faculty of Technology, Bielefeld University, Bielefeld, Germany; ^2^ Center for Biotechnology (CeBiTec), Bielefeld University, Bielefeld, Germany; ^3^ Biochemical Engineering (AVT.BioVT), RWTH Aachen University, Aachen, Germany; ^4^ Institute of Physics, University of Augsburg, Augsburg, Germany

**Keywords:** CRISPR/Cas9, CHO, cell line development, ChIP-seq, epigenetics, safe harbor, stability, histone

## Abstract

Chinese hamster ovary (CHO) cells are the most important platform for producing biotherapeutics. Random integration of a transgene into epigenetically instable regions of the genome results in silencing of the gene of interest and loss of productivity during upstream processing. Therefore, cost- and time-intensive long-term stability studies must be performed. Site-specific integration into safe harbors is a strategy to overcome these limitations of conventional cell line design. Recent publications predict safe harbors in CHO cells based on omics data sets or by learning from random integrations, but those predictions remain theory. In this study, we established a CRISPR/Cas9-mediated site-specific integration strategy based on ChIP-seq data to improve stability of recombinant CHO cells. Therefore, a ChIP experiment from the exponential and stationary growth phase of a fed-batch cultivation of CHO-K1 cells yielded 709 potentially stable integration sites. The reporter gene eGFP was integrated into three regions harboring specific modifications by CRISPR/Cas9. Targeted Cas9 nanopore sequencing showed site-specific integration in all 3 cell pools with a specificity between 23 and 73%. Subsequently, the cells with the three different integration sites were compared with the randomly integrated donor vector in terms of transcript level, productivity, gene copy numbers and stability. All site-specific integrations showed an increase in productivity and transcript levels of up to 7.4-fold. In a long-term cultivation over 70 generations, two of the site-specific integrations showed a stable productivity (>70%) independent of selection pressure.

## 1 Introduction

Chinese hamster ovary (CHO) cells are the most important cell-based system for biopharmaceutical production, accounting for 70% of all recombinant proteins (Wurm, 2004; Walsh, 2018; Oosting, 2019). Despite decades of experience, however, many key parameters of production and cell line development—such as chromosome rearrangements, heterogeneity within cell lines, unpredictable expression levels, and gene silencing - are still not fully understood (Lee et al., 2019). The standard method for generating new CHO production cell lines for biopharmaceuticals is based on random integration of the product gene followed by selection *via* antibiotics or metabolic markers (Bebbington et al., 1992; Hamaker and Lee, 2018). Clones are then separated by single cell cloning or limiting dilution (Jayapal et al., 2007; Lai et al., 2013). This method has been continuously optimized over the years by leveraging our constantly expanding knowledge on transcription, translation, cell metabolism, signal transduction pathways and secretion (Tihanyi and Nyitray, 2021). Intuitive genetic modifications of the cells (e.g., knock-down of lactate dehydrogenase) are now standard (Zhou et al., 2011).

Nevertheless, despite this progress, the development of a high-producer cell line still usually takes between 6 and 12 months—making it both time-consuming as well as labor- and cost-intensive (Jayapal et al., 2007; Lai et al., 2013). This is because the random integration method still only relies on chance to generate the right production cell lines. Random integration also generates clones with different gene copy numbers and integration sites, resulting in heterogeneities in the cell population, both in terms of growth and productivity, and in terms of unpredictable expression levels that can additionally change during cultivation (Lee et al., 2019). This makes it very difficult if not impossible to obtain results that are simultaneously reproducible and predictable (Hefzi and Lewis, 2014).

Making matters even worse, adequate stability and predictability with respect to both product yield and quality are absolute prerequisites for any biopharmaceutical-grade production (Barnes et al., 2003; Noh et al., 2020). A cell line may be said to be stable if, firstly, a homogeneous cell population retains 70% or more of volumetric productivity over 70 generations and, secondly, it exhibits no “clinically meaningful differences” (according to the FDA Orange Book) compared to the reference product as determined by considering its structure, function, purity, chemical identity, and bioactivity (Bailey et al., 2012; Dahodwala and Lee, 2019). Instability within recombinant CHO cells can occur at any or all of the genome, transcriptome, or proteome level as reviewed in Dahodwala and Lee (2019). The term “position-effect” is frequently used to refer on the one hand to rearrangements of chromosomes that lead to loss or silencing of genes, and on the other hand to chromatin-related effects caused by different integration sites (Wilson et al., 1990; Recillas-Targa, 2006). Chromosome rearrangements (Derouazi et al., 2006; Du et al., 2013; Bandyopadhyay et al., 2019) or gene copy loss (Kim et al., 2011; Beckmann et al., 2012) have both been identified as potential reasons for instability. Other studies have also indicated that instability may arise from gene silencing (Chusainow et al., 2009; Yang et al., 2010; Osterlehner et al., 2011; Marx et al., 2018), which can be caused by DNA methylations that occur at the promoter (Wippermann and Noll, 2017)—frequently involving the CMV (cytomegalovirus) promoter, specifically (Romanova and Noll, 2018). Based on this, stable clones were screened *via* DNA methylation at the CMV promoter subsequent to random integration, which, however, resulted in a non-negligible false positive rate (Osterlehner et al., 2011). In addition, histone modifications in the environment of the GOI (gene of interest) influence transcription. Some of these modifications and their relevance for gene expression are well elucidated (Kundaje et al., 2015). For example, H3K4me3 is associated with active promoter regions (Lawrence et al., 2016), H3K27ac is associated with increased activation of enhancer and promoter regions (Karlić et al., 2010) and H3K9me3 is a typical marker for constitutive, permanent heterochromatin and is associated with gene silencing (Nicetto and Zaret, 2019). Like CpG methylation, histone modifications were also analyzed to screen for stable expressing cells after random integration. Here, acetylation of H3 and also the appearance of H3K4me3 at the CMV promoter under selection pressure was shown to be a more effective indicator of stable cells than CpG methylation (Moritz et al., 2016).

In order to improve cell line development with respect to stability, reproducibility as well as product yield and quality, one of the most important research interests is on site-specific integration of product genes and the development of faster and more efficient selection and screening systems during long-term cultivation (Tihanyi and Nyitray, 2021). One optimization approach is the specific integration of product genes into what are known as “safe harbors” or “hot spots”. These are regions in the genome that are supposedly not affected by gene silencing and show increased transcriptional activity. At present, very few such safe harbor regions have been identified in CHO cells (Hamaker and Lee, 2018). Their characteristics are poorly understood and differ substantially between the relatively few known regions (Lee et al., 2019). Notwithstanding those limitations, however, it has been shown that site-specific integration into known safe harbor regions can lead to stable cell lines with high productivity (Kawabe et al., 2018; Zhao et al., 2018; Pristovšek et al., 2019).

**FIGURE 1:**
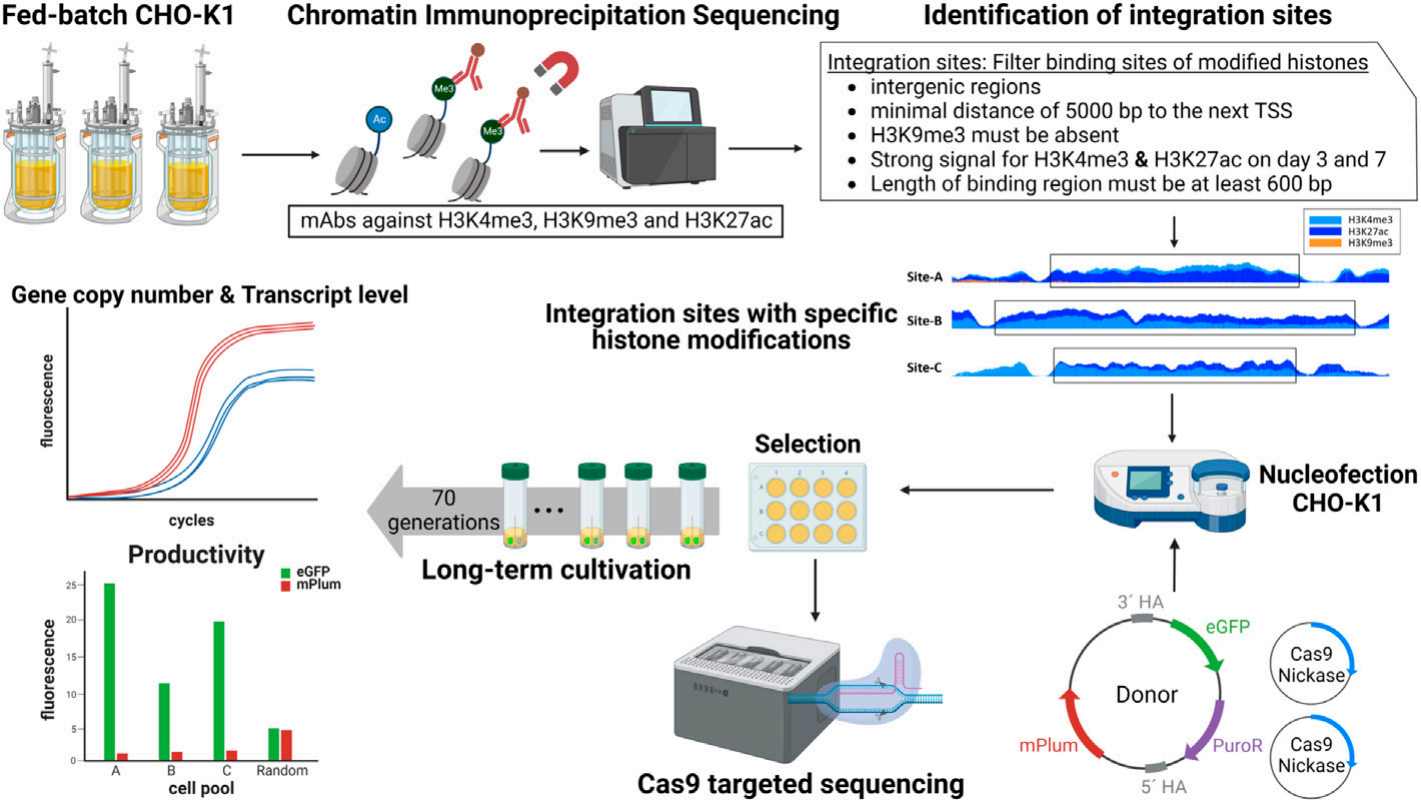
Overview of the workflow for the identification and analysis of site-specific integration into regions with specific histone modifications for enhanced transgene stability.

To date, the ongoing efforts to identify additional safe harbor regions in the CHO genome has mostly been conducted *via* empirical methods such as random lentiviral integrations (Gaidukov et al., 2018; O'Brien et al., 2018; Zhou et al., 2019). Recently, Lee et al. (2019) described a pipeline using omics methods to search for safe harbors in a structured manner. Initial approaches have also used RNA-Seq data to compare properties of stable and unstable integration sites to predict safe harbors (Pristovšek et al., 2019; Dhiman et al., 2020). Another approach sought to identify potential safe harbor regions based on the presence or absence of 16 epigenetic markers, in particular, histone modifications and RNA-Seq data (Hilliard and Lee, 2020). To date, all of these methods have remained theoretical in nature—meaning that they have not actually been tested *via* site-specific integration. Here, we establish a CRISPR/Cas9-mediated site-specific integration strategy based on chromatin immunoprecipitation (ChIP)-seq data that improves the stability of recombinant CHO cells. Figure 1 provides an overview of the identification and analysis of the integration sites with specific histone modifications. We set out in this work to establish *via* proof-of-concept that stable integration sites can be accurately predicted with epigenetic data. Therefore, a ChIP experiment was conducted against H3K4me3, H3K27ac, and H3K9me3, using samples taken from the exponential and stationary growth phase of a fed-batch cultivation of CHO-K1 cells. After genome-wide analysis of histone modifications, analysis of the observable combinations of these epigenetic markers (in broad intergenic regions) was used as a criterion for the identification of potential integration sites. To analyze the influence of histone modifications on transgene expression, the reporter gene eGFP (enhanced green fluorescent protein) was integrated by CRISPR/Cas9 into three regions harboring specific modifications. The site-specific integration was then verified and characterized by targeted Cas9 nanopore sequencing. Subsequently, the cells with the three integration sites to be screened were compared in terms of transcript level, productivity, and stability against a randomly integrated donor vector.

## 2 Materials and methods

All primers for PCR, qPCR, and RT-qPCR, vector construction, and all guide RNAs, are listed in Supplementary Table S1 and were purchased from Metabion.

### 2.1 Cell culture

The CHO-K1 cell line (strain ATCC 61-CCL) was adapted for growth in suspension and used as our model host cell line. CHO-K1 cells were cultivated in shake flasks (Triforest) or TubeSpin® Bioreactors (TPP) at 185 rpm (maximal deflection 50 mm), 37 °C, 5% CO_2_, and 80% humidity within either a Mytron cell culture incubator (Memmert) or in 2 L B-DCU bioreactors (Sartorius). To determine viable cell density (VCD) and viability, cells were counted using a Cedex™ automated cell counter device (Roche). The cells were passaged and diluted to 3 × 10^5^ cells/mL every second to third day for preculture and long-term cultivation. All cultivations took place within the chemically defined TCX6D medium (Xell AG) that was supplemented with 8 mM glutamine.

### 2.2 Bioreactor operation

Bioreactor cultivation was performed as triplicates in 2 L Biostat B-DCU bioreactors (Sartorius AG), with a starting volume of 1 L and an inoculation of 5 × 10^5^ cells/mL. The cultivation temperature, pH-value, and dissolved oxygen concentrations were controlled at 37°C, at 7.2, and 40% of the air saturation, respectively. Stirring speed was set to 120 rpm in the beginning, before being increased to 150 rpm on day 3 and then to 180 rpm on day 6, using a Rushton turbine. The feed (1 L in total) was added, in pulses, on days 3–6 of the cultivation. Beginning with 100 ml, the feed volume was increased by 100 ml every day using CHO Basic Feed (Xell AG), supplemented with 40 mM of glutamine.

### 2.3 Chromatin immunoprecipitation

Samples of 1 × 10^7^ cells were harvested on days 3 and 7 after inoculation of the bioreactors. The SimpleChIP® Enzymatic Chromatin IP Kit (Cell Signaling Technology) was used for the whole ChIP workflow. All steps were performed according to the manufacturer’s protocol. After resuspending fixed cell pellets, the biological replicates were pooled. To digest the chromatin, 5.64 µL micrococcal nuclease were used for each pooled sample. Lysis of nuclei was performed with the Sonifier 250 (Branson) on level 2, in 4 cycles of 5 s. Between cycles, the samples were incubated on ice. Chromatin was stored at -80°C for further analysis. DNA was extracted from 50 µl chromatin solution and the concentration was determined using Nanodrop One (ThermoFisher). The size of the digested chromatin was analyzed with a 1% agarose gel. Samples with a predominant size range between 100 and 500 bp were used for further analysis. Immunoprecipitation was performed with chromatin solution equal to 7.5 µg DNA and 2–10 µl antibody overnight. The following ChIP validated antibodies were used: Tri-Methyl-Histone H3 (Lys4) (C42D8) Rabbit mAb (#9751, Cell Signaling Technology), Acetyl-Histone H3 (Lys27) (D5E4) XP Rabbit mAb (#8173, Cell Signaling Technology), Tri-Methyl-Histone H3 (Lys9) (D4W1U) Rabbit mAb (#13969, Cell Signaling Technology), Histone H3 (D2B12) XP Rabbit mAb (ChIP Formulated) (#4620, Cell Signaling Technology) and Normal Rabbit IgG (#2729, Cell Signaling Technology). All antibodies were used according to the manufacturer’s protocol. Two percent of the starting volume of chromatin were stored at -20°C for normalization of the sequencing data as input sample. ChIP eluates and the input sample were checked *via* PCR using DreamTaq DNA polymerase (Thermo Fisher Scientific), according to the manufacturer’s protocol, with primers specific to *Rpl30* suited for micrococcal nuclease digested DNA.

### 2.4 ChIP-sequencing and data analysis

Library preparation of the ChIP eluates and input samples was performed using TruSeq ChIP Library Preparation Kit—Set A (Illumina). Sequencing was carried out using one NextSeq 500/550 High Output Kit v2.5 (75 cycles, Illumina). Reads with a minimal length of 25 nt after trimming of the sequencing adapters were used for further analysis. Quality control of raw reads was conducted by FastQC v0.11.8 (Andrews 2010). Reads were aligned to the *Cricetulus griseus* Genbank assembly GCA_003668045.1 using Bowtie v1.2.3 (Langmead et al., 2009), with the parameters --*best --strata -m 1*. Alignment statistics were checked using samtools v1.10.2 (Li et al., 2009), and cross-correlation analysis was performed using the run_spp.R script from Phantompeakqualtools package (Kharchenko et al., 2008; Landt et al., 2012). Peaks were called using MACS2 v2.2.6. (Zhang et al., 2008; Feng et al., 2012), with the parameters *callpeak -g mm -- extsize 147 --nomodel -B*, and with the additional parameter *--broad* added for H3K9me3. Differential peak analysis between days 3 and 7 was performed using MACS2 v2.2.6 (Zhang et al., 2008; Feng et al., 2012), with the parameters *bdgdiff -C 11* for H3K4me3 and H3K27ac data, and with *-C* 4 added for H3K9me3*.* The parameter *-C* was used to keep only the strongest peaks for the identification of integration sites. The common peaks were annotated using HOMER 3.12 (Heinz et al., 2010) with the gtf file from the Genbank assembly used for alignment.

### 2.5 Identification of integration sites

The annotated outputs of ChIP-seq data analysis—which contained the strongest common peaks of both times—were used as input for the identification of possible integration sites. These were filtered to keep only intergenic peaks with a minimal distance of 5000 bp to the next TSS (transcription start site). These peak lists were converted to bed format. To keep only regions with both activating histone marks (H3K4me3 and H3K27ac), and no repressive histone marks (H3K9me3), Bedtools intersect v2.27.1 (Quinlan and Hall 2010) was used to first join peaks of activating histone marks and then to perform an anti-join with all peaks of H3K9me3. Overlapping peaks or neighboring peaks with a distance less than 150 bp were merged using Bedtools merge v2.27.1 (Quinlan and Hall, 2010). Possible integration sites shorter than 600 bp were removed. The integration sites bed file was converted to fasta format using the getfasta module from Bedtools v2.27.1 (Quinlan and Hall, 2010). Designing gRNAs for the identified possible safe harbor regions with the CHOPCHOP website (https://chopchop.rc.fas.harvard.edu/), the parameters Chinese hamster ovary, knock-in and CRISPR-Cas9 nickase were used (Labun et al., 2019). The gRNAs with the highest efficiency score were chosen (Table 1). Six gRNAs were used in this study (underlined are the PAM sequences):

**TABLE 1:** gRNAs for site-specific integration into three regions with specific histone modifications using Cas9 nickases.

Site-A		CCAGCT​TAG​GGC​CAC​GCT​GCT​GC	GCG​AGC​ATC​TTG​GAA​GGG​CGGGG
Site-B	TGA​CAA​CAA​CCG​CTT​GCC​AGAGG	CCACGC​TAT​TAG​AAA​ACC​TGC​TC
Site-C	CCCCAT​GAT​AGA​GGT​TCC​GAC​CT	GCT​TCA​TTT​GGC​TGG​CTA​GAAGG

Vectors carrying the gRNAs, and eGFP-Cas were designed for all three integration sites. Single strand oligonucleotides with *Bbs*I overhangs were synthesized by Metabion, phosphorylated and annealed. The gRNAs, were ligated to the digested (*Bbs*I) plasmid pX461 (Ran et al., 2013).

### 2.6 Donor plasmid construction

To investigate the potential of the integration sites to serve as potential safe harbor regions, the reporter gene eGFP was introduced to the identified regions for analysis of expression and stability. The donor plasmids consist of site-specific 5′ and 3′ homology arms (about 800–850 bp each), protein expression cassette [hCMV-promoter, eGFP, bGH Poly(A)], puromycin expression cassette [SV40-promoter, puromycin, bGH Poly(A)], mPlum expression cassette [hCMV-promoter, mPlum, bGH Poly(A)], and an Ampicillin/ori backbone. The plasmids were cloned *via* classical restriction and insert cloning into an in-house plasmid. The comparts of the plasmids were then amplified using Q5 or Phusion High Fidelity DNA Polymerase (both NEB), according to the manufacturer’s protocol. Finally, the plasmids were purified using the Miniprep Kit NucleoSpin Plasmid or the Midiprep Kit NucleoSnap Plasmid (both Macherey-Nagel), and sequenced at the Sequencing Core Facility (CeBiTec, Bielefeld University).

### 2.7 Generation of stable cell pools

For the generation of stable cell pools, 2 × 10^6^ CHO-K1 cells were co-transfected with the corresponding CRISPR- and donor-plasmids (a total amount of 6 µg DNA, equally divided to each plasmid) with the Lonza Nucleofector 2b system (Lonza), using the Amaxa Cell Line Nucleofector Kit V (Lonza) and program U-023, according to manufacturer’s instruction. Two days after transfection, the cells were treated with 4 µg/ml puromycin for selection. The transfected cell pools were monitored by flow cytometry (BioRad S3e Cell Sorter) to determine the eGFP and/or mPlum positive cell populations during the selection phase.

### 2.8 Isolation of genomic DNA

Genomic DNA was isolated from 5 × 10^6^ cells and purified using the Wizard Genomic DNA Purification Kit (Promega), according to the manufacturer’s protocol for Tissue Culture Cells and Animal Tissue.

To isolate high molecular weight DNA from 5⋅10^6^ cells for nanopore sequencing, the NucleoBond HMW DNA Kit (Macherey-Nagel) was used as outlined in the manufacturer’s instructions.

### 2.9 Targeted Cas9 sequencing and data analysis

For targeted Cas9 sequencing the Cas9 Sequencing Kit (SQK-CS9109) (Oxford Nanopore Technologies) was used according to the manufacturer’s instructions. For each sample, 5 µg genomic DNA were used as input. The DNA was then dephosphorylated to block untargeted adapter ligation and cleaved at the eGFP target site using Cas9 and custom crRNAs (Integrated DNA Technologies). Blunt ends resulting from Cas9 cleavage were then dA-tailed and ligated to sequencing adapters. Sequencing was carried out on the GridION MK1 using one R9.4.1 flow cell per sample without barcoding.

Fastq files from each flowcell containing reads that passed the quality filtering were concatenated. To identify reads that originate from the target sequence, these were mapped to the eGFP sequence using Minimap2 v2.17 (Li, 2018) with the parameter *-ax map-ont*. Alignment statistics were checked, and mapped reads were extracted, using samtools v1.10.2 (Li et al., 2009). Sam files with extracted reads were converted to fastq format using the SamToFastq module of Picard Toolkit (Broad Institute, 2019). These targeted reads were aligned to the *Cricetulus griseus* Genbank assembly GCA_003668045.1 using Minimap2 v2.17 (Li, 2018) with the parameter *-ax map-ont.* Alignments marked as supplementary or with MAPQ score smaller than 20 were discarded. The enrichment of eGFP integrations achieved by targeted sequencing compared to the theoretical expected number of eGFP integrations considering the sequencing depth and gene copy numbers was calculated.

$$fold\;enrichment=\frac{reads\;mapping\;to\;eGFPintegration/2}{gene\;copies\;eGFP*\left(\frac{bases\;sequenced}{bases\;CHO\;genome}\right)}$$

The start of the genomic alignments was compared to the intended integration site and then classified as either site-specific or random to calculate the integration specificity. This was done using a custom R script.

### 2.10 RNA Isolation

For total RNA extraction, cell suspension equal 5 × 10^6^ cells were centrifuged for 7 min at 800 *g*, 4°C. Cell pellets were then resuspended in 600 µl TRI Reagent (Zymo Research), and stored at -80°C. The lysed samples were mixed with 120 µl chloroform and were centrifuged at 12,000 *g*, 4°C for 15 min. The aqueous phase was extracted again with 300 µl TRI Reagent and 120 µl chloroform. RNA was precipitated from the aqueous phase by incubation with the same volume isopropanol and centrifuged at 12,000 *g*, 4°C for 10 min. The RNA pellet was washed with 600 µl 75% ethanol twice. After drying, the pellet was resuspended in 50 µl RNase-free water.

### 2.11 Analysis of relative gene copy numbers and mRNA levels

The relative *eGFP* transgene copy numbers and mRNA levels were determined *via* qPCR analysis. *eGFP*, glyceraldehyde-3-phosphate dehydrogenase (*GAPDH*), β-2-microglobulin (*B2m*), β-actin (*ACTB*), and vezatin (*VEZT*) primers were designed and tested for specificity. To determine gene copy numbers of *eGFP* relative to *GAPDH* and *B2m*, 1000 ng isolated DNA was combined with 15 µl GoTaq qPCR Master Mix (Promega), 0.6 µl forward and 0.6 µl reverse primer (10 µM), and then replenished with water up to a total volume of 20 µl. The analysis was carried out with the LightCycler® 480 (Roche) in triplicates for each sample. The following thermal cycling parameters were applied: 10 min at 95°C followed by 40 cycles of 15 s at 95°C, 30 s at 60°C and 30 s at 72°C. A melting-curve was generated after the last cycles from 50°C to 97°C. For mRNA level detection relative to *ACTB* and *VEZT*, the Luna Universal One-Step RT-qPCR Kit (NEB) was used according to manufacturer’s instructions with 500 ng of RNA for each sample. The measurement was done in triplicates with the LightCycler® 480 (Roche).

The relative quantifications were determined by the analysis of crossing point (CP)-values with the second derivative maximum method. All results were normalized to an inter-run calibrator for possible variation in input amount and quality of DNA or RNA between samples. Relative amounts were calculated by pairing the target gene with both reference genes (pairing rule: all to mean). Afterwards, both the mean and standard deviations were calculated.

### 2.12 Determination of fluorescence signal

For the determination of the fluorescent signal, 5 × 10^6^ cells were first centrifuged for 5 min at 200 *g*, 4°C, then washed, and then centrifuged again for 7 min at 400 *g*, 4°C. The cell pellet was then frozen at -80°C. The frozen pellet was then lysed with 1 ml of lysis buffer (Supplementary Table S2) supplemented with PMSF (Phenylmethylsulfonylfluoride), cooled down on ice, and homogenized by ultrasonic treatment (Branson 1210 Ultrasonic Cleaner, Emerson Electric) for 5 min. After being cooled on ice for 30 min, the samples were centrifuged for 20 min at 15,000 *g*, 4°C. The fluorescence signal was analyzed in triplicates with 100 µl of the supernatant each using the TECAN-Reader Infinite 200 Pro MNano+ and corrected by the autofluorescence of the cells. The excitation of eGFP (emission 507 nm) was measured at 488 nm, the excitation of mPlum (emission 590 nm) at 649 nm.

## 3 Results

### 3.1 Genome-wide analysis of histone modifications for the identification of integration sites

The host cell line CHO-K1 was cultivated in a chemically defined medium in 2 L bioreactors and fed with a commercial feed solution. Samples for ChIP-Seq were cross-linked and frozen during the exponential growth phase on day 3 (viability >95%), before feeding and the stationary growth phase (92% viability) 1 day after the last feed pulse (Supplementary Figure S1). ChIP-Seq was performed for three of the six core histone modifications according to the International Human Epigenetics Consortium (Kundaje et al., 2015). Sequencing yielded 34.9–58.0 M uniquely mapping reads, with a mean quality of 34.4 and a length of 83 bp. Cross-correlation analysis resulted in a normalized strand cross-correlation coefficient (NSC) > 1.05 and a relative strand cross-correlation coefficient (RSC) > 1.8 for narrow peaks which corresponds to a quality tag (Phantompeakqualtools) of two. The quality tag for broad peaks was one.

**FIGURE 2:**
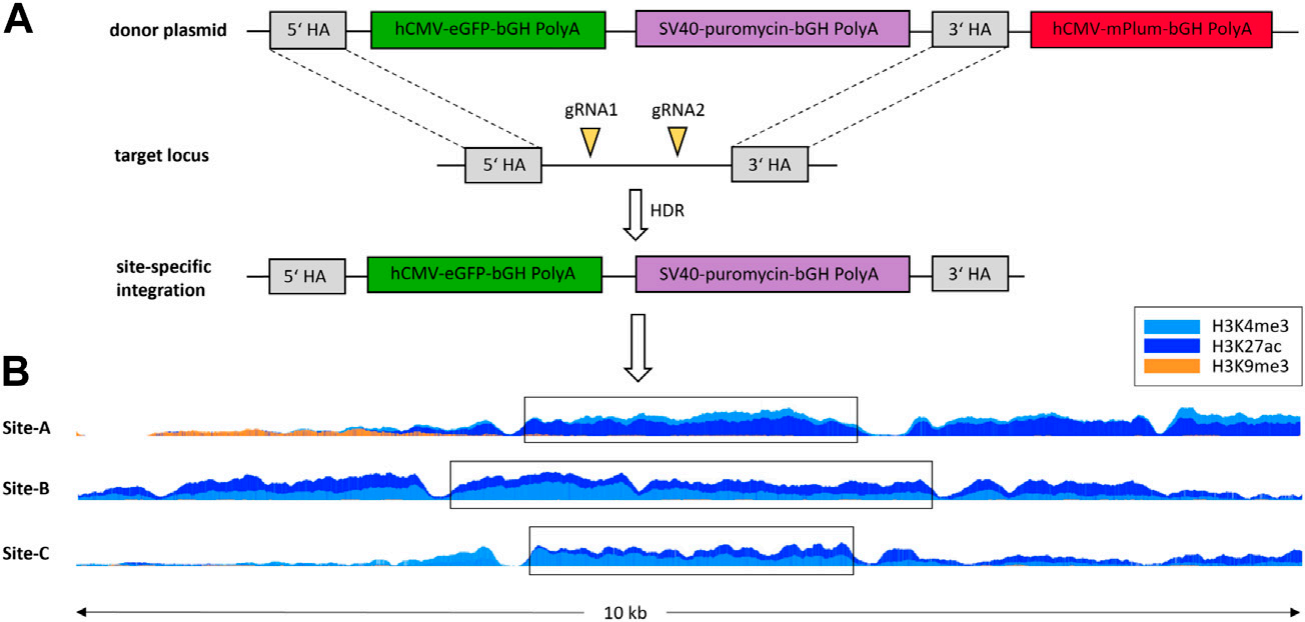
Schematic illustration of site-specific integration of eGFP-PuroR-cassette without mPlum *via* HDR (homology directed repair) **(A)** into regions with specific histone modifications. The pileup of histone modifications in a 10 kb window around the tested integration sites **(B)**.

Regions with favorable histone modifications for the stable expression of a transgene should meet the following criteria: high levels of both H3K4me3 and H3K27ac, and low levels of H3K9me3, at both time points in a region with a minimum width of 600 bp. These regions were not allowed to overlap loci expressing coding RNAs or regions 5000 bp upstream of the associated TSS. Application of this criteria resulted in the identification of 709 possible integration sites with specific histone modifications. The pileup at the three tested integration sites is shown in Figure 2. In all integration sites, H3K9me3 is absent. Integration site A has H3K4me3 as dominant histone modification, while sites B and C have a higher occupancy of H3K27ac. The integration sites are all between 2600 and 3900 bp wide.

### 3.2 Identification of site-specific integration and verification using targeted Cas9 sequencing

**FIGURE 3:**
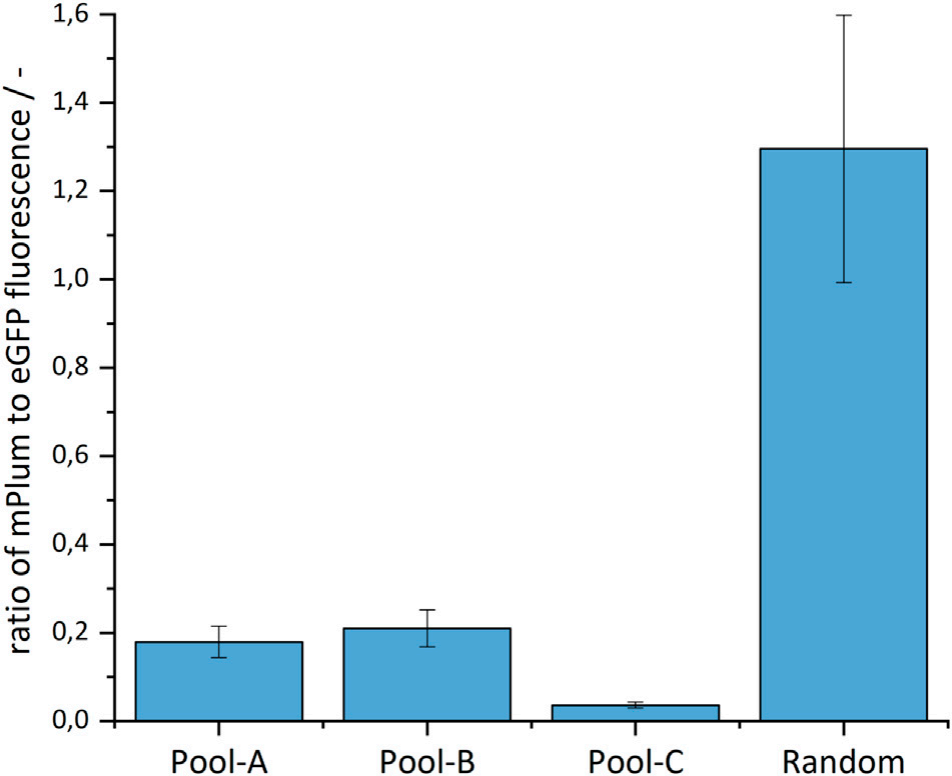
Ratio of the mPlum to eGFP fluorescence signal in the site-specifically and randomly integrated cell pools.

Following Sergeeva et al. (2019), a sequence for an additional fluorescent protein - the mPlum - was located on the plasmid outside the homologous arms. This served as a marker for the type of integration. Through random integration, both eGFP and mPlum should integrate (resulting in green/red fluorescent cells), whereas in site-specific integration, only the eGFP should be stably integrated into the genome (resulting in only fluorescent green cells, Figure 2A). As shown in Figure 3, the ratio of the mPlum to eGFP fluorescence signal in the site-specifically integrated cell pools was between 0.05 and 0.2 at the end of the selection phase. The ratio of the random cell pool was approximately 1.3. This ratio provided a first indication of the specificity of the CRISPR experiment.

The ratio of mPlum to eGFP fluorescence provides a strong preliminary signal for the type of integration—but sequencing is necessary to provide confirmation. In addition, the specificity of the integration must also be assessed. Since PCR errors should be avoided and long reads are needed to span the insert, homologous arms and integration site, Targeted Cas9 Sequencing from Oxford Nanopore Technologies was used. Compared to other Cas9-based target-enrichment sequencing protocols, no capture step is required and read length is limited solely by the fragment length of the DNA (Slesarev et al., 2019; van Haasteren et al., 2021).

From each sample, 33500–56300 mappable reads (with an average length of ∼5 kb) were sequenced. This is equal to 6–17% of the CHO genome. The integration site must be enriched, in order to facilitate detection, because only 26–57 reads are assigned to both the insert and the genome (which corresponds to an on-target rate of 0.15–0.33% at base level). Compared to the theoretically expected number of inserts in the sequencing runs—normalized to the gene copy number—targeted reads are accordingly enriched 86–244x with an average length of ∼12.6 kb.

**FIGURE 4:**
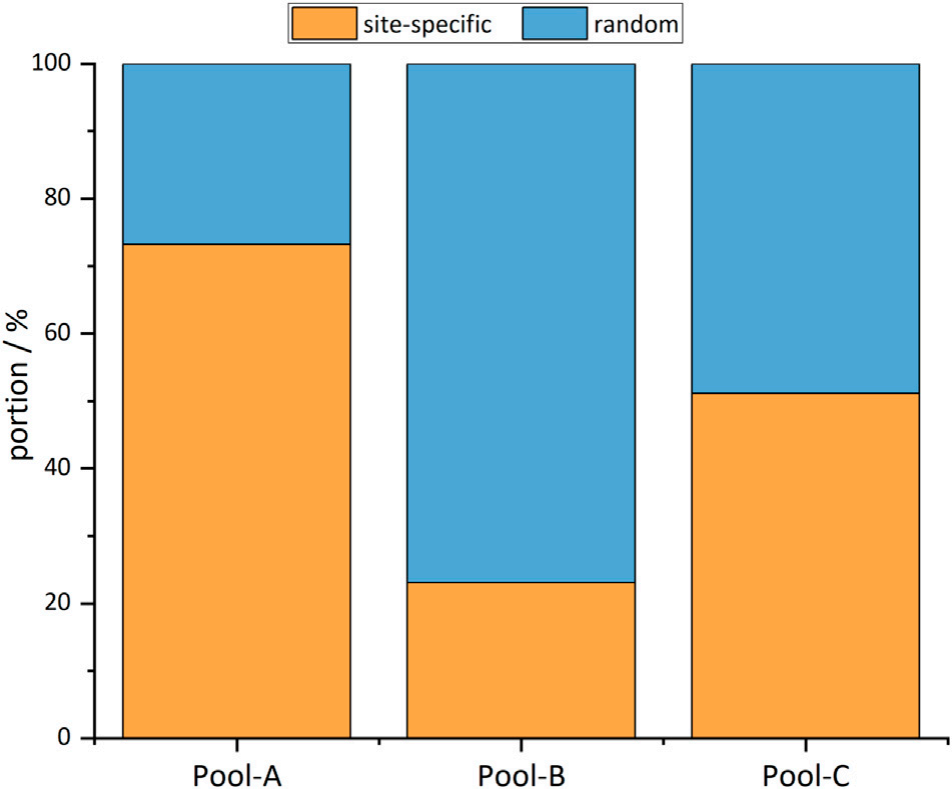
Portions of reads mapping to the intended integration site (site-specific) and random locations.

The comparison of the alignments with the intended integration sites shows that site-specific integration took place in all cell pools. However, random integration also occurred to a different extent in all pools (Figure 4). The proportion of site-specific integration is identical with the specificity: Pool-A displayed an integration specificity of 73%, Pool-B of 23%, and Pool-C of 51%. In most cases, site-specific integration occurred at exactly one location; however, in all pools, there were integrations in the same regions but with an offset of up to 900 bp. In the following measurements, cell pools with a mixture of site-specific and random integration are considered. The unspecific integration seems to be truly random and should therefore perform like the random cell pool.

### 3.3 Analysis of transcript levels and productivity

**FIGURE 5:**
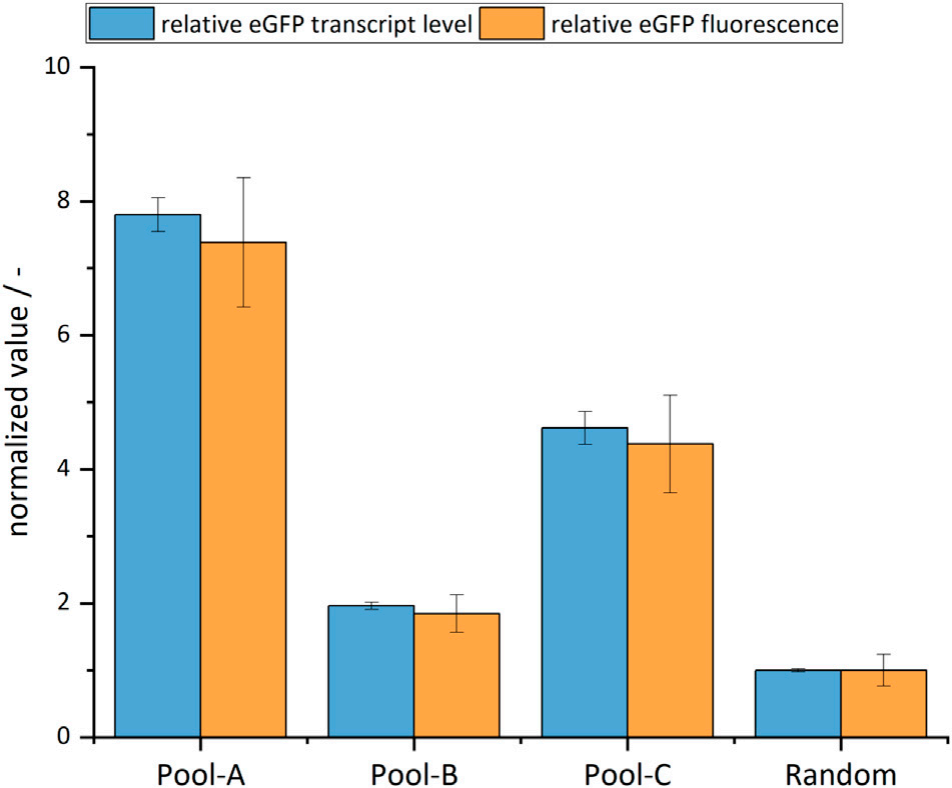
Relative transcript levels and fluorescence of eGFP of site-specific cell pools and the random cell pool set to 1. The error bars represent the standard deviation of the technical replicates.

After successful integration of eGFP into regions with specific histone modifications, the first step was to compare productivity and transcript levels with random integration. Cell pools were sub-cultured with selection pressure for 3 weeks, and the eGFP fluorescence intensity was then measured. The transcript level of eGFP was analyzed at the same time point *via* RT-qPCR, in order to ensure that any increased productivity could be correctly attributed to transcription rather than translation. The results are shown in Figure 5.

It was found that all site-specifically integrated cell pools showed a higher fluorescence intensity when compared with random integration. Specifically, Pool-A showed an increase by a factor of 7.4 (±0.97), Pool-B by a factor of 1.9 (±0.97), and Pool-C by a factor of 4.4 (±0.73). The transcript levels also showed almost the same fold changes compared to the random integration. There was a strong correlation between transcript levels and eGFP fluorescence (Pearson r=0.99). Compared to the specificity of integration, the productivity data indicated a similar relationship. This suggests that productivity in monoclonal cell lines would be even higher, and at a similar level, in all site-specifically integrated cell pools. In summary, both the productivity and transcript levels of all site-specifically integrated cell pools were higher than those of the randomly integrated one.

### 3.4 Enhanced Transgene Expression Stability

To evaluate the stability of eGFP production, the three site-specifically integrated cell pools and the random cell pool were cultivated for 49 days with (4 µg/ml puromycin) and without selection pressure. Fluorescence intensities and gene copy numbers were analyzed at the beginning (T0), in the middle (T21) and at the end (T49) of this long-term cultivation period.

In all long-term cultivations performed, the viability of the cultures was above 95% over the entire cultivation period, regardless of the addition of selection pressure. No differences were observed in the cell densities and growth rates that were achieved between any cultures at the same time. An increase in the specific growth rate *μ* was observed equally in all cultures over the cultivation period. This rate was assessed once a week, and observed over a period of 3 days. The specific growth rate *μ* initially averaged 0.90 days^−1^, and increased to an average of about 1.05 days^−1^ by the end of cultivation. Similar observations of increased growth rates for CHO cells with increasing number of passages were also noticed in other long-term cultivations (Kaneko et al., 2010; Beckmann et al., 2012).

**FIGURE 6:**
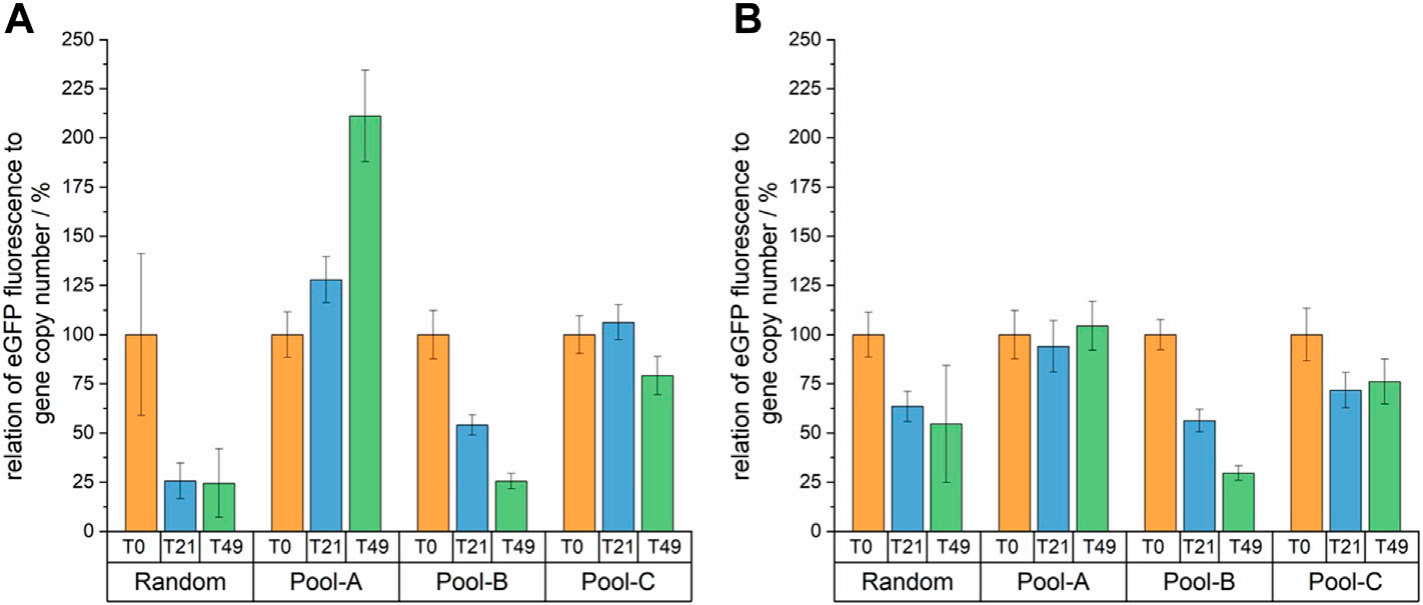
Relative eGFP fluorescence normalized to the gene copy number of days 0, 21 and 49 of a long-term cultivation with **(A)** and without **(B)** selection pressure. All values are shown relative to T0.

To determine the stability of eGFP production, fluorescence intensities were normalized to gene copy numbers. The results are shown in Figure 6. In the progress of cultivation clear differences began to manifest due to the decreasing fluorescence intensities of the random cell pool–and by day 21, an increased fluorescence intensity could be detected for all site-specifically integrated cell pools compared to the randomly integrated cell pool. After 49 cultivation days, however, this could no longer be observed for cell pool B, as the fluorescence intensity of this cell pool decreased noticeably. For Pool-C, only a slight decrease in normalized fluorescence intensity was observed. For Pool-A, the intensity remained constant at about 120 without selection pressure and increased from about 175 to about double with selection pressure, presumably due to a selection on a subpopulation with strong fluorescence.

The criteria for the stability of a homogeneous cell line are the retention of 70% of the productivity over 70 generations, as well as the maintenance of “clinically relevant” properties such as structure, function, purity, chemical identity, and bioactivity (Dahodwala and Lee, 2019). As eGFP is not a clinically relevant product, this aspect cannot be assessed for this experimental approach. In addition, it should be noted that the present cultures are all cell pools, rather than homogeneous cell lines.

Nevertheless, it was observed that Pool-A remained stable over time in relation to productivity without selection pressure (97 ± 13% of productivity is maintained) and that productivity even increased under selection pressure (211 ± 31%), due to selection to high fluorescent cells. This translates to a 15-fold increase in productivity after 49 days compared to the random integration. For Pool-C, 79 ± 14% with puromycin and 72 ± 14% without puromycin of the fluorescence intensity were preserved. If the standard deviations are not included, then this cell pool also fulfilled the stability criterion—although it should be noted that only about 51% of the cells corresponded to the desired site-specifically integrated cells. In a homogeneous cell line, the maintenance of productivity would have been possibly even higher, as the decrease was probably largely due to silencing in the random integration sites. The same considerations should also be kept in mind when looking at the percentage fluorescence intensity decrease of Pool-B. After 70 generations, the fluorescence intensity was only 26 ± 5% and 31 ± 4% of the initial value. Pool-B can thus not be described as stable. However, since only about 23% of the cells were site-specifically integrated, it is possible that a monoclonal cell line with this integration site could also be stable.

As expected, the fluorescence intensity of the random cell pool decreased from day 0 to day 49. This confirmed the typical problem of instability over time for CHO cell lines. Since the relative gene copies were determined by qPCR, and did not change over time, a loss of gene copy number can be excluded as a reason for the instability in this case.

## 4 Discussion

In terms of biopharmaceutical production, CHO cells represent the most prevalent platform—and despite decades of experiments, many parameters of cell line development (like expression levels and gene silencing) still remain very difficult to predict. Nevertheless, this study represents a proof-of-principle that safe harbors can be predicted using ChIP-Seq with a minimal set of histone modifications.

For this purpose, three histone modifications were analyzed genome-wide from a CHO-K1 bioreactor fed-batch cultivation. The criterion for possible integration sites was defined as the intergenic occurrence of the modifications H3K4me3 and H3K27ac, and the absence of H3K9me3. Using this method, 709 potential integration sites with these specific histone modifications were identified. Compared to other safe harbor predictions based on a combination of multiple omics datasets (Dhiman et al., 2020; Hilliard and Lee, 2020), the method presented here can be much more readily adapted to individual host cell lines, as a single cultivation run with sequencing on a single flowcell is sufficient. Three integration sites were examined for expression strength and stability, only one (B) of which matched the landing pads predicted by Dhiman et al. (2020).

For integration, the CRISPR/Cas9 system was used. A donor vector coding for eGFP was site-specifically integrated. Outside the homology arms was the gene coding for mPlum, a second fluorescent protein, located. Thus, cells with site-specific integration displayed only green fluorescence, while cells with random integration displayed both green and red fluorescence. This double-fluorescence system was particularly useful during selection, showing an mPlum:eGFP ratio of 0.05–0.2 for site-specifically integrated cell pools and 1.3 for random integration. However, the system did not provide quantitative information about the specificity and selectivity of integration—so Cas9 targeted nanopore sequencing (Gilpatrick et al., 2020) was used first-in-CHO. To be able to identify the integration-sites, the method was adapted by inverting the crRNAs. With this technique, no PCR amplification and capture step is needed, and the read length is limited solely by the length of the DNA fragments. An enrichment of 86–244-fold was achieved with a mean read length of 12.6 kb. Sequencing showed that site-specific integration was achieved in all cell pools, but the specificity differed. Pool-A had a specificity of 73%, Pool-B 23% and Pool-C 51%. Thus, after limiting dilution, in the worst case about every fourth cell would show the desired integration, which would make the search for strongly and stably expressing cells unnecessary. By this, weeks could be saved in cell line generation, and it would no longer rely on chance. Surprisingly, in Pool-A an integration of mPlum into the desired integration site could also be detected in a few reads.

Until now, predictions of safe harbors from omics data sets have remained theoretical. Therefore, the described cell pools were directly used for the analysis of transcription, productivity and stability. An initial evaluation of the eGFP transcript levels and fluorescence showed an increase of 1.9–7.4-fold compared to the random integration without taking into account the specificity of the integration. Both analyses showed a high correlation for all cell pools. Even more important in this context is maintaining productivity of at least 70% over 70 generations (Dahodwala and Lee, 2019). To investigate this, the cell pools were sub-cultivated with and without selection pressure for 49 days (equivalent to 70 generations). In this period the specific growth rate increased from 0.9 days^−1^ to 1.05 days^−1^ in all cultures. The same effect was previously observed in other CHO long-term cultivations (Kaneko et al., 2010; Beckmann et al., 2012). The eGFP fluorescence was measured over time and normalized to the gene copy number to analyze the silencing effect independent from the loss of gene copies. Random integration and Pool-B, which had the lowest integration specificity, are both unstable over the period analyzed. Pool-A and -C, on the other hand, maintain their productivity both with and without selection pressure. Under selection pressure, the productivity of Pool-A even doubles over time. Part of the effect could be explained by the fact that resistance to puromycin in cells with undirected integration is epigenetically silenced over time and they die. A similar increase in productivity over time was observed by Zhao et al. (2018) after site-specific integration into C12orf35 locus.

The strategy to integrate transgenes into regions with high levels of H3K4me3, H3K27ac and low levels of H3K9me3 in intergenic regions improved the development of stable cell lines significantly without altering the growth behavior. Constructing a stable recombinant CHO cell line takes between 6 and 12 months using conventional random integration (Lai et al., 2013). With the method presented in this study, targeted transfected cell pools with mostly intended integration-sites can be created in 3 weeks. After limiting dilution, just a handful of cells must be screened to obtain a stable cell line. Moreover, no laborious and time-consuming screening for integration sites by random integration is necessary for the identification of safe harbors (Zhou et al., 2019), but only a single ChIP sequencing experiment from one cultivation run.

## Data Availability

The sequencing data presented in this study are deposited in the NCBI SRA, NCBI BioProject PRJNA865478.
