# Supplementary material for: Enhancing stability of recombinant CHO cells by CRISPR/Cas9-mediated site-specific integration into regions with distinct histone modifications
Source: Front Bioeng Biotechnol. 2022 Oct 13;10:1010719. doi: 10.3389/fbioe.2022.1010719 (PMC9606416; doi:10.3389/fbioe.2022.1010719)
Supplement: Supplementary file 1 [file DataSheet1.docx]

Supplementary Material

# Supplementary Figures and Tables

Supplementary tables and figures are provided below. A complete list of identified integration sites with specific histone modifications is provided in a separate Excel file.

## Supplementary Figures
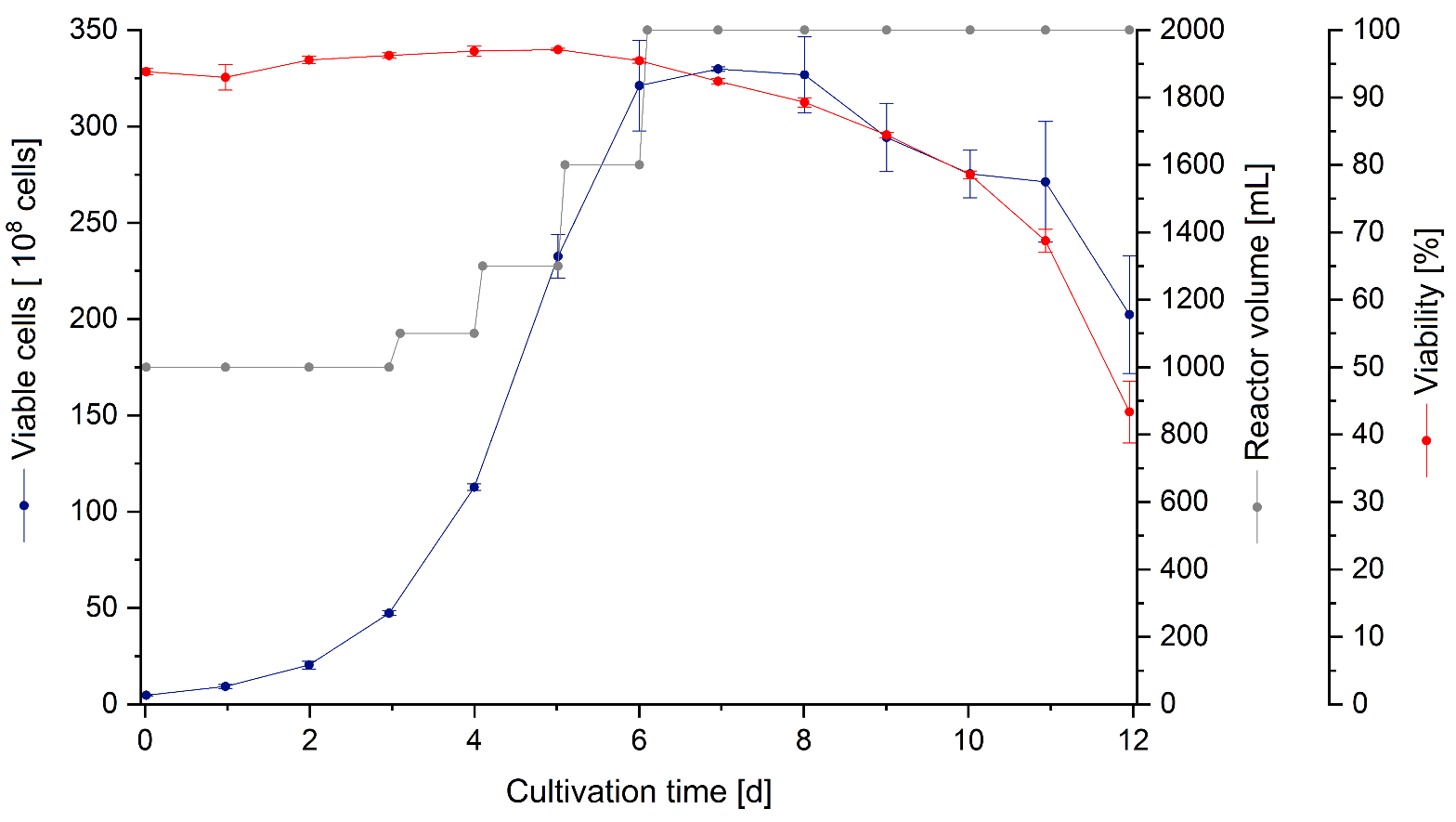


Supplementary Figure 1: Viable cells, viability and volume of CHO-K1 bioreactor fed-batch cultivations. The error bars represent the standard deviation of three parallel experiments

## Supplementary Tables

Supplementary Table 1: Sequences used for PCR, qPCR, RT-qPCR, gRNA cloning and targeted CAS9 sequencing.

| **Name** | **Sequence 5´ 🡪 3´** |
| --- | --- |
| eGFP_Nukl_2_for | ACCCTCGTGACCACCCTG |
| eGFP_Nukl_2_rev | GGCGGACTTGAAGAAGTCGT |
| refB2m_qPCR_fw | CTT GGG CTC CTT CAG AGT GG |
| refB2m_qPCR_rev | TGG ACA AAG TCG AGC TGT CA |
| refGapdh_qPCR_fw | CTG GTA TGT GGG ATC AGA AAC AGT |
| refGapdh_qPCR_rev | CAT GAG GTC CAC CAC TCT GTT G |
| eGFP_qPCR_for | GGC AAG CTG ACC CTG AAG TT |
| \|  \| eGFP_qPCR_rev \| \| --- \| --- \| | GGC GGA CTT GAA GAA GTC GT |
| ACTB for cDNA | CAC CCT GTG CTG CTC ACC |
| ACTB rev cDNA | CGT ACA TGG CTG GGG TGT |
| VEZT for cDNA | TTA AGG AGC TGG GGC TTG |
| \|  \| VEZT rev cDNA \| \| --- \| --- \| | GTG CCA CCC AGA GTT GGA |
| ChIP_RPL30_for | GGT TTA AGA AGC TTA CCT GGG TC |
| ChIP_RPL30_rev | GCA CAG CAT GTG GGA AAT ACT AC |
| crRNA_ eGFP_plus (Seq) | ATCCGCCACAACATCGAGGA |
| crRNA_ eGFP_minus (Seq) | CGATGCCCTTCAGCTCGATG |
| K4_7_gen_for | TTCCCTCAAATTCCCAGCGG |
| K4_7_gen_rev | TCTAAGGAGATGCGTCCGGG |
| K27_132_gen_for | ACACTGGAGAAGGGCCAATG |
| K27_132_gen_rev | AGCCCAGGTAGACCAGATGT |
| K27_4247_gen_for | TGCTTTGCCTACCTGGTCTC |
| K27_4247_gen_rev | TTTTGGGTGATGTCTGGGCT |
| K4_7_5HA_for | CGTACGGTACCcTAGTGGAACTTGTGGGTCTcg |
| K4_7_5HA_rev | TTACGCTCGAGAGCTGGGCTAAGtgggaac |
| K4_7_3HA_for | CTGGATTAATTAAGCGGGGGTGTGGATGCTACC |
| K4_7_3HA_rev | GCTAAACGCGTTCCCTCCGGTCTAGGGCTCTT |
| K27_132_5HA_for | CTAAGGGTACCacctctccctccctcctta |
| K27_132_5HA_rev | TACGCTCGAGCAGAGGATAAGGACGGACAAGG |
| K27_132_3HA_for | GATCTTAATTAAGCGTGGAAGGAGATTTGACA |
| K27_132_3HA_rev | CTAAGACGCGTtctagatccgTCAGGTTGACA |
| K27_4247_5HA_for | TCTTAGGTACCccaggcccagcctgGCG |
| K27_4247_5HA_rev | ATTCCTCGAGGATAGAGGTTCCGACCTGGGGC |
| K27_4247_3HA_for | CTTGTTAATTAAAGCCAGCCAAATGAAGCAAA |
| K27_4247_3HA_rev | GGTACACGCGTTTGGACTGCAGTTGACTGTGG |

Supplementary Table 2: Composition of lysis buffer.

| **Concentration** | **Substance** |
| --- | --- |
| 50 mM | Tris-HCl pH 7.2 |
| 150 mM | NaCl |
| 2 mM | EDTA |
| 0,1 % (w/v) | SDS |
| 1 % (v/v) | NP40 |
